# What hinders the uptake of computerized decision support systems in hospitals? A qualitative study and framework for implementation

**DOI:** 10.1186/s13012-017-0644-2

**Published:** 2017-09-15

**Authors:** Elisa G. Liberati, Francesca Ruggiero, Laura Galuppo, Mara Gorli, Marien González-Lorenzo, Marco Maraldi, Pietro Ruggieri, Hernan Polo Friz, Giuseppe Scaratti, Koren H. Kwag, Roberto Vespignani, Lorenzo Moja

**Affiliations:** 1 Cambridge Centre for Health Services Research (CCHSR), Department of Public Health and Primary Care, University of Cambridge School of Clinical Medicine, Forvie Site, Robinson Way, Cambridge, CB2 0SR UK; 2 Unità di Epidemiologia Clinica, IRCCS Istituto Ortopedico Galeazzi, Via Riccardo Galeazzi 4, 20161 Milan, Italy; 3 Dipartimento di Scienze Biomediche per la Salute, Università degli Studi di Milano, Via Carlo Pascal 36, 20133 Milan, Italy; 4 Dipartimento di Psicologia, Università Cattolica del Sacro Cuore, Largo Agostino Gemelli 1, 20123 Milan, Italy; 5 Dipartimento di Scienze Biomediche per la Salute, Università degli Studi di Milano, Via Carlo Pascal 36, 20133 Milan, Italy; 6 Clinica Ortopedica, Università degli Studi di Padova, Via Giustiniani 3, 35128 Padova, Italy; 7 Dipartimento Internistico, Ospedale di Vimercate, Via Santi Cosma e Damiano 10, 20871 Vimercate, Italy; 8 Medical School of International Health, Ben Gurion University of the Negev, P.O. Box 653, 84105 Beersheva, Israel; 9 IRST Istituto Scientifico Romagnolo per lo Studio e la Cura dei Tumori IRCCS, Via Piero Maroncelli 40, 47014 Meldola, Italy

**Keywords:** Clinical decision support systems, Health information technology, Evidence-based medicine, Qualitative research, Healthcare professions

## Abstract

**Background:**

Advanced Computerized Decision Support Systems (CDSSs) assist clinicians in their decision-making process, generating recommendations based on up-to-date scientific evidence. Although this technology has the potential to improve the quality of patient care, its mere provision does not guarantee uptake: even where CDSSs are available, clinicians often fail to adopt their recommendations. This study examines the barriers and facilitators to the uptake of an evidence-based CDSS as perceived by diverse health professionals in hospitals at different stages of CDSS adoption.

**Methods:**

Qualitative study conducted as part of a series of randomized controlled trials of CDSSs. The sample includes two hospitals using a CDSS and two hospitals that aim to adopt a CDSS in the future. We interviewed physicians, nurses, information technology staff, and members of the boards of directors (*n* = 30). We used a constant comparative approach to develop a framework for guiding implementation.

**Results:**

We identified six clusters of experiences of, and attitudes towards CDSSs, which we label as “positions.” The six positions represent a gradient of acquisition of control over CDSSs (from low to high) and are characterized by different types of barriers to CDSS uptake. The most severe barriers (prevalent in the first positions) include clinicians’ perception that the CDSSs may reduce their professional autonomy or may be used against them in the event of medical-legal controversies. Moving towards the last positions, these barriers are substituted by technical and usability problems related to the technology interface. When all barriers are overcome, CDSSs are perceived as a working tool at the service of its users, integrating clinicians’ reasoning and fostering organizational learning.

**Conclusions:**

Barriers and facilitators to the use of CDSSs are dynamic and may exist prior to their introduction in clinical contexts; providing a static list of obstacles and facilitators, irrespective of the specific implementation phase and context, may not be sufficient or useful to facilitate uptake. Factors such as clinicians’ attitudes towards scientific evidences and guidelines, the quality of inter-disciplinary relationships, and an organizational ethos of transparency and accountability need to be considered when exploring the readiness of a hospital to adopt CDSSs.

**Electronic supplementary material:**

The online version of this article (10.1186/s13012-017-0644-2) contains supplementary material, which is available to authorized users.

## Background

Computerized Decision Support Systems (CDSSs) are a type of software that aims to support clinical decision-making by connecting health professionals with evidence from high-quality scientific research. Advanced CDSSs link patient-specific information in electronic health records (EHR) with evidence-based knowledge to generate case-specific guidance messages through a rule- or algorithm-based software [1, 2]. Innovation in the content and format of information has led to sophisticated and large-scale guidance: computer aids cover a wide spectrum of critical decisions, including treatment dosing, interactions, and complex situations where the balance between benefits and harms is subtle, such as thrombolysis for acute ischemic stroke [3] and pulmonary embolism [4].

Though evidence of the benefits of CDSSs on clinical outcomes is still limited, a systematic review suggests that CDSSs may reduce morbidity by 10–20% [1]. More evidence exists on the impact of CDSSs on process outcomes, such as appropriateness of medication prescribing, early detection of adverse drug reaction, use of preventative care, and access to accurate medical records [2, 5–8]. Most studies have been conducted in medical settings, but preliminary evidence suggests that CDSSs may improve care in surgical fields too—for example by preventing venous thromboembolism in surgical patients [9, 10].

Despite this promising evidence, studies consistently show that the mere provision of CDSSs in clinical settings does not guarantee their uptake [11–17]. Even where CDSSs are readily available, clinicians often ignore their alerts and fail to adopt their recommendations [12]. Understanding the reasons behind these behaviors is essential for effective implementation of CDSSs.

### Existing theories on the uptake of information technology and scientific evidence

CDSSs are a prime example of a complex healthcare intervention characterized by “a number of components, which may act both independently and inter-dependently” [18, 19]. CDSSs combine at least two such components: (1) the adoption of a new health information technology and (2) the attempt to integrate clinical evidences into routine care processes (i.e., knowledge translation).

Interventions designed to facilitate the uptake and spread of healthcare information technologies have been analyzed by a variety of theoretical approaches, including, but not limited to, those outlined in Table 1 (see [20–23] for a more comprehensive overview). Theories inspired by ergonomics and human factors engineering, including the study of technology design and evaluation, have traditionally focused on the *usability* of new technology*—*i.e., the perceived ease and fluency of use of the technology by its potential users [24]. Most studies on the uptake of CDSSs focus on such usability issues, identifying the excessive number of alerts triggered by CDSSs or the users’ lack of access to personal computers or tablets as the most prominent barriers to use [11, 13, 14, 25].

**Table 1:** The main approaches to the study of technology uptake

Theoretical approach	Disciplinary roots	Example of theories	Focus
Usability	Ergonomics, Human Factors Engineering, Human-Computer Interaction, Information System	Usability [49, 50], Task Technology Fit [51], GOMS models [52]	The use and spread of a new technology depends on the usability and learnability of the technology itself. Understanding the interactions between technology and its end users is key to improve usability.
Technology acceptance	Cognitive-Behavioral Psychology, Behavioral Theories	Technology Acceptance Model (TAM) [26], TAM2 [27], Unified Theory of Acceptance and Use of Technology (UTAUT) [28]	Focuses on the predictors of individuals’ intention to adopt a technology. Technology acceptance is determined by (a) users’ perceived usefulness (degree to which a person believes that using a technology would enhance job performance); (b) users’ perceived ease of use (degree to which a person believes that using a particular system would be free from effort). Subsequent models introduced social influence as further determinant of technology acceptance.
Organizational theories	Organizational and management studies, Organizational Psychology, Organizational Development	Organizational justice [53], Leadership theories [54], Organizational culture [55]	Focuses on the organizational barriers and facilitators to the uptake and spread of technologies, such as the integration of technologies into existing systems and workflow, management commitment to the new technology, the presence of a structured program for implementation, the presence and quality of training.
Practice theories	Sociology, Anthropology, Social Psychology, Philosophy	Normalization Process Theory [56], Technological sense-making [57, 58], Sociotechnical Systems [59], Actor Network Theory [60], Communities of Practice [46]	Technology adoption and spread is conceived as a *social practice*, constantly produced and re-produced through people’s actions-in-context. What people do (e.g., technology adoption) is informed both by what they know about a certain technology and their situated local judgments about the meaning of such technology in their (social and material) context.

Technology acceptance theories [26–28] place more emphasis on users’ expectations and characteristics and propose that the key determinants of technology adoption are the *ease of use* and *perceived usefulness* of the technology itself, with *social influence* added as a third determinant in subsequent models [27]. Organizational and practice theories both highlight the need to go beyond a focus on technology acceptance by isolated individuals to look at actions and judgments *in context* [23, 29–31], including cultural and professional norms, organizational infrastructures, and broader social and political influences (e.g., policies, laws, and regulations). With a few exceptions [16, 17, 32], concepts from organizational and practice theories have rarely been mobilized to explain the uptake of CDSSs, although available frameworks for IT implementation confirm their importance [33].

CDSSs are not just a new information technology: they have a specific knowledge-translation function. CDSSs “materialize” the evidence-based paradigm, integrating external evidence with the course of action of clinicians; this may add further barriers to CDSSs’ uptake. We know that health professionals sometimes discard the use of evidence out of fear of compromising their critical reasoning, medical judgments, and professional autonomy [34–36] or because scientific evidences challenge deep-rooted hierarchies and systems of power based on seniority [15, 37]. These barriers vary broadly across countries and medical settings, but evidence suggests that they continue to shape knowledge mobilization and quality improvement interventions globally [38, 39]. While CDSSs have been envisaged as a medium to *overcome* these barriers [40], little attention has been devoted to the fact that clinicians’ resistance to the use of scientific evidence may, in fact, hinder CDSSs’ uptake.

Studies of CDSSs in the context of emergency medicine found that users’ trust in CDSSs (e.g., the extent to which CDSSs were perceived to be “risk averse”) and the quality of inter-occupational relationships influenced the use of the technology [16, 17]. Clinicians’ attempts to defend their professional autonomy and expertise have also been found to influence their perceptions and use of CDSSs [32, 34]. These important findings, however, have not been linked with the specifics of the implementation process: studies have mainly been conducted in contexts in which CDSSs had already been introduced, while the perceived facilitators and barriers existing prior to CDSS introduction, or for the evolution of such perceptions throughout the technology’s various stages of uptake, remain largely unexplored.

The current qualitative study examines the barriers and facilitators to the uptake of an evidence-based CDSS as perceived by diverse health professionals in hospitals at different stages of CDSSs’ adoption. Using the knowledge generated by our empirical data, and drawing insights and sensitizing concepts from existing theories on technology and evidence adoption, it aims to construct a framework for guiding the implementation of CDSSs.

## Methods

### Protocol

The protocol of this study has been previously published [25]. This nested qualitative study, inspired by a Grounded Theory (GT) approach [41], is part of a mixed-method research project including two pragmatic randomized controlled trials testing the effectiveness of MediDSS—an evidence-based CDSS that produces patient-specific, point-of-care reminders (Table 2) [42, 43]. The trials are currently being conducted. The intervention represents an advanced type of CDSS: support rules are activated and embedded in the electronic patient records, provide advice on diagnosis or medication or laboratory test decisions, and cover hundreds of medical conditions.

**Table 2:** The evidence base of MediDSS

The CDSS evaluated by the trials is named “MediDSS” (Medilogy Decision Support System) and is produced by Medilogy, an Italian IT company. MediDSS is the Italian translation of Evidence Based Medicine Electronic Decision Support (EBMeDS). This is a CDSS developed by Duodecim Medical Publications Ltd., a company led by the Finnish Medical Society Duodecim, marketed in several countries [61]. EBMeDS is derived from EBMGuidelines, which is a web-based compendium of the best available evidence on treating a wide range of common conditions. EBMeDS complies with the pillars of evidence-based medicine: the service implements an active literature search to identify current relevant information, privileges a cumulative versus discretionary approach (i.e., prioritization of systematic reviews over other evidence sources), critical appraisal, adopts a formal grading of evidence, and clearly differentiates citation of expert opinions from other evidence sources in summaries [62, 63]. The quality of these services, including their evidence-based nature, has been repeatedly evaluated.	

### Settings and participants

We collected data in four Italian hospitals that differed in their level of information technology integration and access to evidence-based guidelines. Setting A was an orthopedic hospital relying entirely on paper-based clinical documents; it did not have an EHR or a CDSS. Setting B was an orthopedic research hospital that abandoned paper-based clinical documents to adopt an EHR in 2011 but had not yet adopted a CDSS. Setting C1 was an oncology hospital that adopted an EHR in 2008. Since 2015, the hospital’s EHR has been linked to a variety of CDSSs, including evidence-based messages on treatment and diagnosis using care management algorithms. Setting C2 was a general hospital with similar characteristics to C1, featuring an EHR linked to advanced CDSSs. Table 3 summarizes the features of each setting.

**Table 3:** Settings

	Setting A	Setting B	Setting C1	Setting C2
Type of hospital	Orthopedic hospital	Orthopedic research hospital	Oncology research hospital	General hospital
EHR	No	Yes	Yes	Yes
CCDS	No	No	Yes	Yes
Trial setting	No	No	Yes	Yes

Participants included end users of the CDSS (doctors and nurses) as well as the other actors that play in important role in shaping the structural and political underpinning of CDSSs’ adoption, such as information technology (IT) staff and members of the hospital board of directors. Including these individuals in our sample allowed us to examine not only clinicians’ willingness and ability to use a new technology but also the impact of a broader cultural and political initiative to make medical decisions less discretional [21, 35]. The final sample (*n* = 30) is summarized in Table 4.

**Table 4:** Participants

	Setting A	Setting B	Setting C1	Setting C2	Tot
Doctors	3	3	3	4	13
Nurses	2	2	2	2	8
Managers	2	1	1	1	5
IT staff	1	–	2	1	4
Total	8	6	8	8	30

The sampling criteria were defined a priori [25]. However, consistently with the GT principle of “theoretical saturation” [41], the final number of research settings and interviewees-per-role was decided in the course of data collection, based on preliminary analysis of a sub-sample sample of interviews. For example, setting C2 was not included in the original study sampling; it was added during data collection to explore further complexity in the experiences of using CDSSs. Similarly, since the views and accounts of nurses were more consistent with each other than those of doctors (surgeons and physicians), we decided to expand our sample to include more doctors than nurses.

### Data collection

We collected data using semi-structured interviews. The interview guide was informed by our literature review and sensitizing concepts and focused on (a) participants’ views of and experiences with health information technologies, (b) specific beliefs and experiences with CDSSs, (c) values attached to scientific evidence and willingness to adopt clinical recommendations in routine practice, and (d) perceptions regarding the potential of CDSSs to integrate evidence and guidelines in clinical practice. The topic guide was used flexibly and adapted to the different professional roles. For example, while clinicians were prompted to reflect on their first-hand experiences of clinical information technologies and CDSSs, hospital managers were asked to discuss the hospital high-level strategies with respect to the same topics. Managers and IT staff were also encouraged to reflect on potential ways to tackle clinicians’ resistance towards CDSSs. No individual refused to take part in the study. Audio-recorded interviews were 45-min long, on average, and conducted in the workplace between February 2014 and December 2016. Informed consent was obtained by all participants.

### Data analysis

**Fig. 1:**
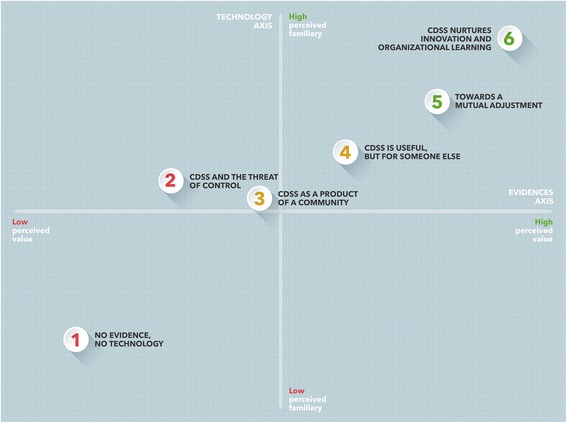
The six positions represent different degree of perceived control over CDSSs. Each position is characterized by a degree of perceived control over, and mastery of, information technologies and scientific evidence. Since progression through the position is not necessarily linear, the figure should be interpreted as an indicative, rather than definitive, representation of the process of CDSSs’ uptake

We used a constant-comparison, GT-inspired approach [41, 44] to analyze the interview transcripts. We started by carefully reading the interviews from each setting, to ensure that they were each understood in terms of their own context. We then performed a line-by-line coding, which involved the matching of each emerging theme with a specific code that was created inductively to relay the experiences described (example of these codes are reported in Additional file 1). We constantly compared data across settings in order to identify reasons for their differences and similarities. In doing so, we did not find a univocal, linear relationship between settings’ structural characteristics (level of information technology integration and access to evidence-based guidelines) and participants’ attitudes towards CDSSs. However, participants’ *perceived* familiarity with information technologies and their *perceived* value of, and trust towards, scientific evidences did play an important role in shaping clinicians’ views of the CDSS. By gradually aggregating and refining and our preliminary codes, and plotting them on a framework composed by the two above dimensions (familiarity with technology and trust towards evidences (Fig. 1)), we identified six consistent clusters of experiences of the CDSS, which we named “positions.” The six positions represent a gradient of acquisition of control over the CDSS (from low to high). The concept of *negotiation of control* over the CDSS is the “core category” of our analysis. Additional file 1 summarizes the stages of the coding process.

Crucially, the choice of organizing our findings through “positions” (rather than a static list of facilitators and barriers) allowed us to advance our understanding of the *process* of CDSSs’ uptake as explained to us by the interviewees. It also enabled us to capture the complexity offered by our sample and to contradict the hypothesis that participants’ views of CDSSs were solely influenced by the structural characteristics of their context: participants from the same setting often provided a variety of different views, condensed into different “positions” (see Additional file 2). We emphasize that our framework should not be intended as a linear/chronological model of CDSSs’ process of adoption: the positions are not necessarily progressive, nor do they overlap with necessary implementation phases. Rather, they serve as an analytical tool to give (presentational) order to participant’s reflections and characterize the various types of barriers to CDSSs’ uptake.

## Results

The uptake of CDSSs can be described as a process of *negotiation of control* between the system and its users. This process is articulated into six “positions,” i.e., coherent clusters of experiences of, and attitudes towards, CDSSs, as described by our participants. Different types of barriers and facilitators to CDSSs use are prevalent in each position.

The first two positions are characterized by clinicians’ low perceived control over CDSSs and include the most severe barriers to CDSSs’ uptake. Clinicians’ concern that the CDSS may reduce their professional autonomy or may be used against them in the event of medical-legal controversies is represented in these two positions. The more clinicians gain a sense of control over the CDSS (as captured by the last positions), the more the obstacles described above are substituted by technical and usability problems. Since these barriers do not impinge on professionals’ power, status, and identity, they are likely to be easier to tackle with sufficient technical support.

In Fig. 1, the six positions are organized along two axes: (1) the perceived value of, and trust towards, scientific evidences (horizontal axis, oriented from left to right) and (2) the perceived familiarity with information technologies (vertical axis, oriented from bottom to top). Each circle represents a specific position. Differences in the degree of perceived control over the CDSS are expressed by the progression of colors from red to green.

### Position 1. Doctors as artisans: no evidence, no technology

*The actual evidences in [orthopedic surgery] are not very many, you know, I can’t really see how [the CDSS] would be useful for us. (…). The actual tools of an orthopedic resemble those of a crafts worker. [...] We learn by reading books and articles, but also by (…) observing the experts at work, learning how they do things... (Physician, setting A)*

In this position, clinicians do not seem to consider the CDSS as a useful working tool in their setting, and adoption is perceived as unlikely. In some cases, the main barrier is related with the perception of scientific evidence. Evidence is negatively viewed as generalized as abstract knowledge, incapable of practical implementation in clinical practice. Engaging pragmatically with the “art and craft” of medicine, and learning from the “eminences” in the field, seems to represent a more conventional course of action. It is worth noting that none of our participants identified directly with these views, but some (especially in setting A) attributed them to some colleagues.

In other cases, obstacles to the uptake of CDSS are associated with the threat posed by the introduction of a new information technology. It is feared, for example, that the EHR would modify existing working practices and alter inter-professional relationships and communication. Moreover, as found by previous studies [34, 45], the EHR is perceived has having a negative effect on doctor-patient communication during consultations and seen as a potential obstacle to the development of a relationships of trust between clinicians and patients. These barriers resonated with the opinion of some IT staff:

*Physicians are afraid of being turned into bureaucrats. Physicians’ power and authority derives from the diagnostic and therapeutic process: physicians don’t want to take their hands off the patient and sit at a desk to write at the computer, they don’t want feel like they are doing data entry. (IT specialist, setting A)*

In this position, the adoption of the CDSS seems unlikely. Resistance to change is likely to be exacerbated by the absence of an organizational culture supporting the use of scientific evidence and information technology, and by professionals’ attempts to defend their current approach to health care delivery.

### Position 2. Either me or it: CDSS and the threat of control

*I believe in our expertise, our conscience and experience. It’s humiliating to think that we can be substituted by a computer! (…) We need to have the courage to do what we think is right, not to merely comply with the guidelines dictated by a system. (…) The knowledge that I get from visiting 150 patients is more substantial than what (the CDSS) can give me. (Physician, setting B)*

The CDSS is perceived as encroaching upon physicians’ competency and jurisdiction; rather than a useful support to their practice, the CDSS is described as a potential hindrance to the exercise of clinicians’ judgment and critical thinking. Clinicians seem to be left with two opposite options: either rejection or unconditioned adherence.

The fear of losing control over autonomous decision-making becomes particularly acute in the event of medical-legal controversies. Some participants expressed concern that the presence of CDSSs may provide non-medical experts with the formal authority to judge medical decisions, thus forcing physicians to follow the system’s advice to avoid the risk of medical and legal charges. Since the CDSS would reveal the discrepancy between formal guidelines and contextualized decisions, clinicians may become legally vulnerable. Judges, lawyers, or other professionals lacking specific medical expertise may appraise physicians’ actions based solely on the indications of CDSSs, without considering other aspects that guide the clinical decision-making process.

*It’s a double-edged sword. Let’s say I don’t turn it on and for whatever unrelated reason something happens to the patient. A judge may say “How is it that you didn’t use the CDSS even if you could have?” It can be used against us. (Physician, setting B)*

*Some doctors asked me […] what if I do something that is different from what the CDSS suggested? […] So what happens if a doctor refuses to follow the system’s recommendation? […] I think the legal framework needs to be clarified. (IT staff, setting C1)*

Disputes over power and control seem to involve other organizational actors as well. One physician felt that, since the CDSS allows widespread access to scientific evidences, the system might lead to nurses’ control or oversight of medical decisions, thus providing an occasion to renegotiate professional boundaries.

*If for some reason I don’t follow the recommendations of the CDSS, a nurse may notice this and say “According to the CDSS, the doctor made a mistake”. Do you see what I mean? [...] If we want to implement it it’s key to discuss the rules of access for each profession. (Surgeon, setting B)*

CDSSs are perceived as limiting, rather than supplementing, physicians’ competencies, expertise and critical thinking: by making the evidence non-negotiable, they are seen as frustrating clinicians’ expertise and reducing their professional autonomy.

### Position 3. Who controls the controller? The CDSS as the product of a community

*Who controls the controller? I would want to know who puts the evidence into the system, to be sure that it’s reliable. [...] We used to think that medicine should be evidence-based and not authority-based. I think we need good authorities to help us selecting the best available evidence.” (Surgeon, setting A)*

The CDSS is received with a more open mind in this position. Clinical evidence is conceived as the product of a community. The trust towards these communities becomes a central theme, and their credibility and authoritativeness concerns clinicians. The CDSS is no longer seen as an “omnipotent” tool but rather as a human-made product that requires legitimization.

Some participants emphasize the potential danger of CDSSs disseminating poor or not updated evidence. They are concerned that young physicians may become accustomed to following CDSSs’ recommendations without questioning them—an eventuality that would be particularly problematic in the event of outdated evidence. This issue is sometimes referred to as the “prompter effect.”

*It’s this sense of false security given by the CDSS… Thinking “Oh well, the computer will tell me the answer” … And people sort of switch their brains off. […] Especially young physicians […]. CDSSs have an incredible potential, but they do expose us to unexpected risks. (Manager, setting C1)*

Similarly, introducing a new information technology is not seen as a “good” or “bad” choice per se; rather, its success or failure depends on the new system’s quality and ability to integrate existing clinical processes and routine activities. This position reflects an increased perceived control over the CDSS; facilitating the uptake of the CDSS, thus, seems to require legitimization of its designers and sources.

### Position 4. Really useful but not for me: the CDSS as a tool for someone else

*It’s brilliant. Really, really useful. I think it’s more so for medics though, rather than [surgeons]. (Surgeon, setting B)*

*Maybe I could use it. I think it would be more useful for young physicians, those who have only just graduated, or those with little experience... You know, to avoid mistakes... (Senior physician, setting A)*

*I think it would be ideal for general practitioners… More than for us in the hospital. (Physician, setting B)*

This position is characterized by a mismatch between clinicians’ views of the CDSS, described as a valuable and promising tool, and the prospect of its actual use in clinical practice. The CDSS is described as “always more useful for someone else”: hospital professionals point at general practitioners, surgeons at physicians, senior attendants at junior physicians. Some clinicians (especially surgeons) feel that the CDSS may help them only for tasks that are peripheral to their core mandate (e.g., patient post-surgical care rather than the actual surgical operation): since CDSSs would not support them in accomplishing the most valued and rewarding activities, users would not fully exploit the system’s potentiality.

The described mismatch may be caused by clinicians’ lack of familiarity with the CDSS or the lack of participation in its implementation. A hospital manager and a member of the IT staff both suggest that these resistances can be overcome by communicating the CDSS’s benefits in contextual activities, creating opportunities to experience the system firsthand, and involving frontline clinicians in the implementation process (for example, using clinical champions that serve as catalysts for change). Reporting positive implementation experiences and promoting discussion between actual and potential users may also enable bridging the gap between perceptions and actual use.

*Any innovation that has the potential to affect clinical autonomy and decision-making shouldn’t be introduced like an imposition. If it’s perceived as a top-down order, clinicians will reject it. Physicians must agree and engage in the project. Having said that I think a strong endorsement from the top management is essential. (IT specialist, setting C1)*

A manager suggested that, although a number of top-down strategies must be adopted to reach this implementation stage, these are only effective if combined with a constant effort to nurture clinicians’ engagement with, and perception of control over, the CDSS. However, up to this position, the desire to shape the CDSS remains mainly theoretical, lacking practical details for effective changes.

### Position 5. Just a machine… that may actually help you: towards a mutual adjustment

*We see a lot of patients every day, most of them with comorbidities and a lot of medications… It’s not unlikely to make a prescribing error, we are human after all! [...] To have a support that can double check for us, and provide us with updated, ad hoc evidences… I think it’s a blessing!” (Physician, setting C1)*

*We hear things like “The CDSS is just a stupid machine that cannot give me orders!” Well, I think the CDSS is indeed a stupid machine, but that stupid machine can sometimes be crucial to avoid mistakes. (Physician, setting C2)*

The CDSS is perceived as a working tool at the service of its users, which can complement their competencies and skills, rather than threaten their professional autonomy. The role of scientific evidences is fully acknowledged, but physicians are seen as the actual owners of decision-making, and as responsible for contextualizing and applying the evidence to individual cases. Clinicians’ representations of both scientific evidence and information technology create a fertile ground for the uptake of CDSS.

*So this morning we had a patient with hyponatremia, which is notoriously iatrogenic, but because they were taking many medications we were not sure which one caused it. Having the computer at the bedside meant that we were able to consult the evidence right away and identify which of the ten medications they were on was causing the hyponatremia. And we couldn’t have done it without the technological support... (Physician, setting C2)*

Notably, rather than causing disputes over professional boundaries (as in position 2), the CDSS presents an opportunity to foster the type of inter-disciplinary practice that is vital for patient safety; it is suggested, for example, that the system allows nurses to acquire additional control of the appropriateness of prescribing.

*Prescribing is the doctors’ role, but […] if we have a doubt about a prescription, we do ask questions […] and most doctors are OK with this, they don’t perceive it as a challenge. […] The CDSS helps because we are not always familiar with the drugs we administer: it is a quick way to double-check that everything looks OK with the prescription. (Nurse, setting C2)*

In this position, the main obstacles to the adoption of CDSSs involve the usability and technical issues that have been widely explored within literature and insufficient adaptation of CDSSs to local and contextual needs. Frequently mentioned issues include the lack of integration between the EHR and the CDSS’s interface and the fear of experiencing an excessive number of alerts.

*If five alerts pop up every time and you start noticing that most of the times four and a half are useless, you start ignoring them. […] It’s a matter of finding the right balance and to improve the content, to make the alerts actually relevant. (Physician, setting C2)*

### Position 6. The CDSS nurtures innovation and organizational learning

*After we integrated the electronic record we started thinking about how the CDSS could do more than just reporting and actually help us in our practice. For example: if I prescribe an antibiotic for pneumonia, this will never be for less than a few days. […] We can teach the system to give me an alert only when a given time expires. (Physician, setting C1)*

Clinicians are willing to promote the full integration of the CDSS in healthcare contexts. Although none of our participants felt that their hospital had reached this stage, some individuals could anticipate or describe it. The CDSS is perceived as a helpful working tool at the service of clinicians, integrating their skills and critical thinking; IT staff and hospital managers describe it as a milestone of modern hospitals. Strategies are in place to adapt the content of the CDSS to specific clinical contexts in which the technology is implemented, to improve its usability and to address technical problems. The CDSS technological infrastructure follows clinicians’ cognitive flow and paths of action in order to guarantee timely responses and optimal functioning. Clinicians, in turn, are open and willing to “learn” from the CDSS and to innovate their practice in response to the unforeseen opportunities it provides. This *mutual adjustment* between clinical practice, scientific evidences, and a new information technology is iterative and ongoing and runs at the speed of innovation in medicine: it is essential that the CDSS is sufficiently flexible to allow frequent amendments.

*We need to be able to update the CDSS quickly if we want to offer an optimal service to our patients. […] We need to be on top of this because nowadays the guidelines may change more than once every year. (Physician, setting C1)*

Some participants suggest that the availability of CDSSs may foster interdisciplinary work and promote the use of scientific evidence within clinical communities that have been historically excluded from the debate (such as pharmacists and physiotherapists).

*The introduction of the CDSS could have positive cultural and educational effects. It could help us developing a culture of evidence within hospital teams and with the community as well. We could use it… not just individually, but as a tool to get together and discuss complex cases and to monitor some particular patients. (Nurse, setting C2)*

Finally, the CDSS may shape strategic and managerial choices (such as the investment in specific research streams), foster a culture of evidence-based policy, and nurture a culture of collaboration between clinicians, hospital management, and IT personnel, which is likely to be essential to guarantee the success of technology-based improvement efforts.

*I would love to start thinking about decision support systems for our strategic and managerial choices too. Evidences for decision-makers. […] We do a lot of work on [evidence-based policy] but I think we could improve in the way we access this knowledge on a day-to-day basis… The work on CDSS is inspiring us to improve. (Hospital manager, setting C1)*

## Discussion

Our study contributes to the literature in three main ways. First, it emphasizes the need to go beyond an approach focused on optimizing CDSSs’ usability to consider the broader social, cultural, and institutional influences that impact CDSSs’ adoption. Factors such as clinicians’ attitudes towards scientific evidence and guidelines, the quality of inter-disciplinary relationships, and an organizational ethos of transparency and accountability need to be considered when exploring the readiness of a hospital to adopt CDSSs. We echo Pope et al.’s suggestion that CDSSs should be conceived “both as a computer technology *and* as a set of practices related to that technology, kept in place by a network of actors in particular contexts” [16].

Second, this study shows that introducing a CDSS is not an off-on switch; rather, it can be articulated through a process of *negotiation of control* over the CDSS, which is characterized by a different set of barriers depending on the stage of the process. When the CDSS is perceived as an “omnipotent” and threatening tool that interferes with clinicians’ autonomy and critical thinking, uptake is likely to be compromised. The more clinicians gain a sense of control over the CDSS, the more likely they are to perceive the technology as a valuable working tool that can complement their competencies and skills; these views and attitudes correspond to an increased willingness to use CDSSs. This novel way of explaining CDSSs’ uptake—grounded in empirical data, but informed by existing concepts from implementation theories and social sciences—highlights the need to go beyond the provision of a static list of obstacles and facilitators and to keep a watchful eye on the process of implementation of CDSSs.

**Fig. 2:**
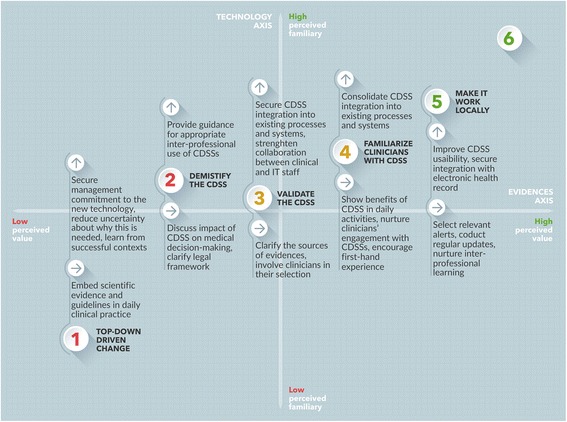
Framework for implementation of CDSSs. The framework describes the actions that, from each position, may facilitate the uptake of CDSS

This brings us to the third contribution of our study: by reflecting on the features of each position and on their mutual relationships, we offer a framework to guide the implementation of CDSSs. Our framework specifies the positive factors in each position that can facilitate CDSSs’ uptake; in so doing, it may help healthcare managers to (1) diagnose the facilitators and barriers that are most relevant to their organizational contexts and (2) prioritize actions and interventions for maximum impact. The framework is discussed below and summarized in Fig. 2.

In the first and second positions, facilitating the uptake of CDSSs requires a process of demystification of the system, addressing preconceived notions and insecurities. Existing studies confirm that the lack of knowledge of a new technology and the reasons for its introduction may hinder adoption, emphasizing the importance of a suitable introduction of the system to the target group [33, 40, 45]. In the case of CDSSs specifically, a crucial step is to clarify the legal framework underpinning its adoption (especially in the event of legal-medical controversies), and to offer guidance on how to manage and report cases of non-compliance with CDSS recommendations. Organizational studies suggest that management commitment to new information technologies and the ability to reduce uncertainty about why this is needed are key predictors of the success of implementation [21, 33]. Senior leaders should, therefore, raise awareness of the actual functions of the CDSS and negotiate the terms and conditions of transparent inter-disciplinary use. Nurturing inter-disciplinary collaboration and an organizational ethos of transparency and accountability is crucial to accomplish this – studies informed by social practice theories may offer guidance [16, 17]. Making substantial IT investments in hospitals where conservatism and hesitation are still prominent may not be efficient nor cost-effective.

In the third and fourth positions, hospital managers may appeal to the positive attitudes shown by some clinicians to overcome the skepticism of others. Social legitimation of the new working tool depends crucially on the perceived control on the CDSS and its level of integration into the hospital workflow. Concepts from both usability theories and social practice theories may be mobilized here to support CDSSs’ adoption. Developing and fostering communities of practice [46] around the design and optimization of CDSSs, including senior and junior clinicians as well as IT staff, may help legitimize the new working tool and enhance the usability of the technological interface. Involving clinicians in the selection of evidence and ensuring transparency in the selection criteria is also key to successful implementation. The implementation of CDSSs should follow a user-centered approach, modulating the relationship between clinicians and rules at the micro-level. “Blanket rules” established at the institutional level that uniformly address all professionals may cause them to ignore the CDSS.

The most challenging obstacles to CDSSs’ adoption are overcome in the fifth and sixth positions. The potential of the CDSS is shown to be fully exploited when the system is used to nurture inter-professional and inter-disciplinary exchanges, and to create a forum in which clinicians can discuss how to best apply scientific evidence to individual patients. The remaining barriers to adoption may derive from a lack of integration of CDSSs into daily practice [45], suggesting that efforts should be directed at increasing the system’s usability and flexibility as well as adaptation to the needs of different users and workflows. It is essential to engage clinicians’ participation in refining existing alerts and to create new ones, ensuring that the CDSS can be easily updated to mirror rapid advancements in guideline development. A participatory approach, however, should not be conceived in isolation from other, more directive, strategies. This is especially true in countries like Italy, where most frontline clinicians do not have first-hand experience with the CDSS or formal training in the development of rules and alerts.

Our framework offers an understanding of the factors that influence the implementation of CDSSs and provides guidance on how to overcome persistent obstacles. It does do not, however, address how change takes place or the causal mechanisms leading to the adoption or non-adoption of CDSSs—hence, it is not, in itself, a theory [47]. Concepts from existing theories (including usability, organizational and social practice theories) are embedded in the framework and are used to qualify the positive forces that, from each position, may facilitate CDSSs’ uptake.

Our findings resonate with existing implementation theories such as Normalization Process Theory [48] that postulates that the way complex interventions become embedded in everyday practice depends on how they interact with existing knowledge, professional relationships, division of labor and the wider organizational context [17]. Our findings are also largely consistent with available implementation frameworks, such as Cresswell et al.’s “Ten key considerations for the successful implementation and adoption of large-scale health information technology” [33]. What our framework adds is an acknowledgement of the complex nature of CDSSs and the two key components they entail (a new information technology and a knowledge translation intervention), which shape specific barriers and facilitators to implementation.

It is important to emphasize that the positions we describe are not necessarily progressive or linear, nor do they represent compulsory phases in the process of implementing CDSSs: the positions should be conceived as analytical tools that can assist in identifying facilitators and obstacles to CDSSs’ uptake, based on the accounts of key stakeholders. The six positions are, conceptually, mutually exclusive; however, a single interview has sometimes contributed to more than one position. Each position attempts to capture the different “voices” in our sample in a way that is sensitive to the frames and interpretations derived by their professional roles. Clinicians’ accounts form the core of each position, reflecting the end users’ view of the CDSS; IT staff and hospital managers, on the other end, assisted in outlining the organizational barriers/facilitators to CDSSs and constructing our framework for implementation.

We have not found a linear correspondence between the structural conditions of different contexts (their level of information technology integration and their access to evidence) and the varying positions each context endorses. For example, structurally “mature” settings (such as C1) feature some of the strong barriers to CDSS such as those described in the second position. However, it is important to note the latest positions (i.e., 5 and 6) are only represented in contexts that currently use a CDSS. This seems to suggest that the most challenging of perceived obstacles may exist prior to the introduction of a CDSS, while the use of the technology itself might alleviate tensions and negative expectations.

Distinguishing between the *pre-existing* enabling factors (e.g., organizational features that appear to facilitate uptake that existed prior to the introduction of the EHR or CDSS) and enabling factors that emerged in the process of use proved somewhat difficult. In most cases, the enabling factors we identified may encapsulate a combination of the two. For example, although the existence of positive inter-disciplinary relations may provide a fertile ground to the uptake of the CDSS, the system itself may work as a catalyst for new inter-disciplinary encounters.

The study is limited in its generalizability. Comparisons with studies conducted in other countries and organizational settings will be necessary to strengthen our conclusions. The introduction and uptake of CDSSs should also be examined longitudinally in order to analyze its long-term professional and organizational effects. Our findings may be used to develop surveys or questionnaires to assess clinicians’ attitudes towards CDSSs more extensively across contexts and countries.

## Conclusions

Inspired by human factor engineering, most studies of CDSSs suggest that, in order for CDSSs to be adopted consistently and effectively, they must be usable. Our study shows that broader social, cultural and institutional influences are at play too. Factors such as the attitude of clinicians towards evidences and guidelines, the quality of inter-disciplinary relationships and the organizational ethos of openness and accountability between professional groups need to be considered when aiming to increase CDSSs’ uptake. Our framework assists in diagnosing the most prominent barriers to CDSSs uptake at different stages of implementation and offers a set of tailored actions to progress towards the technology’s more effective adoption.

## Additional files

Additional file 1: Main phases of the coding process. (DOCX 21 kb)

Additional file 2: Sample of exemplary quotes for each position. (DOCX 26 kb)

## Abbreviations

CDSS: Computerized Decision Support System
EHR: Electronic Health Record
GT: Grounded Theory
IT: Information technology
