# Supplementary material for: What hinders the uptake of computerized decision support systems in hospitals? A qualitative study and framework for implementation
Source: Implement Sci. 2017 Sep 15;12:113. doi: 10.1186/s13012-017-0644-2 (PMC5602839; doi:10.1186/s13012-017-0644-2)
Supplement: Supplementary file 1 — Main phases of the coding process. (DOCX 21 kb) [file 13012_2017_644_MOESM1_ESM.docx]

Appendix 1: Main phases of the coding process.

| **Examples of preliminary codes** | **Views of IT / Scientific evidences** | **Refined codes/ Positions** | ↔ Negotiation of control ↔ | **Settings** |
| --- | --- | --- | --- | --- |
| - Surgery as an art and a craft - CDSS hinders patient-physician relationship - *Eminences* vs *evidences*: mutually exclusive - ‘Data-entry’ perceived as a low-status task or perceived as a resource-consuming task | Low familiarity with IT. Technology may  negatively affect patient-physician  relationship  Low trust in  scientific evidences | *1. Doctors as artisans: no evidence, no technology*  Clinicians do not consider the CDSS as a useful working tool. Adoption is perceived as unlikely. |  | A |
| - Use of CDSS may trigger struggles over boundaries of responsibility/power - The EHR modifies inter-professional communication - CDSS reduces autonomy and critical thinking - Struggles over accountability in the event of medical-legal controversies - CDSS expose doctors to malpractice suits | Technology may create opportunities for  inter-professional struggles    Evidences as non-negotiable entities,  reducing clinician’s autonomy | *2. Either me or it: CDSS and the threat of control*  CDSSs are perceived as limiting, rather than supplementing, physicians’ competencies, expertise and critical thinking. |  | A, B, C1 |
| - Need to establish trusted sources for CDSS - The ‘prompter effect’: clinicians may do what the system says without questioning it - Dialogue between clinicians and IT experts is key to uptake - CDSS must adopt clear criteria for evaluating evidence and grading recommendations | New technologies must be integrated into existing clinical processes  in order to work  Evidences as a ‘human’ product:  valuable, but fallible | *3. Who controls the controller? The CDSS as the product of a community*  The CDSS is a human product that requires approval and legitimization by trusted sources. Increased sense of control on CDSS. |  | A, B, C1, C2 |
| - CDSS could help but only for activities that are considered marginal to their practice - CDSS as a useful tool for junior doctors - CDSS as useful for GPs rather than hospital practitioners - CDSS for physicians but not surgeons | New technologies must be integrated into  existing clinical practice  in order to work  Evidences as integral to clinical  work, but may be limited in certain fields | *4. Really useful, but not for me: the CDSS as a tool for someone else*  Mismatch between clinicians’ views of the CDSS, described as a valuable tool with great potentiality, and the prospect of its actual use in clinical practice. |  | A, B, C1 |
| - CDSS as a valuable tool, aiding human memory - CDSS as ‘safety net’ - Barrier: lack of integration between the EHR and the CDSS’s interface - Barrier: excessive number of alerts - Need to constantly update CDSSs’ content - Clinicians are responsible for contextualizing and applying CDSSs’ alerts to individual patients | Trust that technology can be adapted to  clinicians’ activities  Evidences as integral to clinical work | *5. Just a machine… that may actually help you: Towards a mutual adjustment*  CDSS is working tool at the service of its users, which complements their competencies and skills (rather than challenging their professional autonomy). Main obstacles to adoption are usability/technical issues. |  | C1, C2 |
| - Strategies are in place to adapt the content of the CDSS - Facilitator: collaboration between medical and IT staff - CDSS nurture new clinical communities - CDSS as a learning and education tool - CDSS may stimulate evidence-based policy | IT as milestone of modern hospitals  Evidences as integral to the  healthcare system | *6. The CDSS nurtures innovation and organizational learning*  CDSS nurtures knowledge-sharing, investment in research and collaboration between clinicians, hospital management and IT personnel. |  | C1, C2 |
