# Supplementary material for: What hinders the uptake of computerized decision support systems in hospitals? A qualitative study and framework for implementation
Source: Implement Sci. 2017 Sep 15;12:113. doi: 10.1186/s13012-017-0644-2 (PMC5602839; doi:10.1186/s13012-017-0644-2)
Supplement: Supplementary file 2 — Sample of exemplary quotes for each position. (DOCX 26 kb) [file 13012_2017_644_MOESM2_ESM.docx]

Appendix 2. Sample of exemplary quotes for each position

|  | **Setting A** | **Setting B** | **Setting C1** | **Setting C2** |
| --- | --- | --- | --- | --- |
| **1** | *It’s the EHR that I don’t like very much… The fact that everyone – internists, cardiologists, orthopedics… anyone can access it… It worries me a little. (…) I’ve always written the record in the patient room: I look at their symptoms and write the record meanwhile, and I talk to them, you know? I’m just not used to sit alone in front of a computer to write the notes.* (Physician)  *Physicians are afraid of being turned into bureaucrats. Their power and authority derives from the diagnostic and therapeutic process (…) They don’t want to feel like they are doing data entry.* (IT specialist)  *We haven’t got a regular moment… A systematic way of consulting clinical evidences. This is left to people’s goodwill and desire to be up-to-date.* (Nurse)  *Before we can think of adopting a CDSS we need to create the appropriate cultural conditions… Abandoning a view that “experts” are to be trusted unconditionally. We are working to create culture that legitimizes the more robust scientific evidences (…) So that if and when a CDSS will be introduced, clinicians will trust it.* (Hospital Manager) | Not available | Not available | Not available |
| **2** | *In prescribing, for example, [CDSSs] would be a great help I think… But maybe with time we’ll all become less expert and we’ll not be able to do anything without it… We will get used to push the “prescribing” button. And that would mean a reduction of expertise across our sector.* (Physician)  *Sometime evidences are only used for defensive reasons, like “I do this because there is a study that suggests that it works”. So you feel like you are protected – in a legal sense. But then, you know, sometimes this really doesn’t help that particular patient…* (Hospital manager) | *I don’t think that patient care should be guided simply by an information system... I believe in our expertise, our conscience and experience as clinicians. It’s just humiliating to think that we can be substituted by a computer.* (Surgeon)  *Some doctors are a bit ‘old school’. We sometimes notice that the evidences say one thing and they say something different… But they do have a lot of experience. Where will the truth lie, somewhere in the middle maybe?* (Nurse)  *I think some nurses may be very happy to use the CDSS, but they may not be ready to discuss with… To take a clear stance in those areas that are still a bit grey and unclear… I think some of them would still be more inclined to do what doctors say rather than propose something new, even if this was supported by the CDSS.* (Hospital Manager) | *Some doctors asked me “What if I do something that is different from what the CDSS suggests? […] So what happens if a doctor refuses to follow the system’s recommendation?” […] I think the legal framework needs to be clarified*. (IT specialist)  *I think people need to be educated on how to use [the CDSS]... Because it cannot... it should not substitute doctors’ expertise and experience… It’s the doctor who knows the patient, who has the full picture of what the patient needs – not the CDSS.* (Nurse)  *In some hospitals they’ve developed these amazing decision support systems but are finding them extremely hard to implement because doctors have the feeling that they’re being … how can I say… substituted by these systems… And that’s a real issue.* (Hospital manager) | Not available |
| **3** | *Who controls the controller? I would want to know who puts the evidence into the system, to be sure that it’s reliable. [...] We used to think that medicine should be evidence-based and not authority-based. I think we need good authorities to help us selecting the best available evidence*. (Surgeon) | *It is important to identify rigorous publications, not all evidence is good evidence. [...] I would try to make the system more interactive… for clinicians to be able to add information, to update the system*. (Surgeon)  *I’m not sure I would be able to know what are the best evidence in my field, the trustworthy ones...* (Nurse) | *There’s so much evidence [nowadays] it’s hard to be absolutely certain that the guidelines are based on studies that are statistically and methodologically sound, that are independent from some funding bodies with vested interests*. (Hospital manager)  *The electronic record isn’t always a good thing. It depends on who designs it and how it is done. If engineers and technicians don’t talk to us, they can’t know what we need and how we work and the whole thing may not be usable*. (Physician)  *I don’t think the issue is around technology acceptance here… It’s about building a trust towards the contents of the CDSSs and those who select and manage these contents. We manage the technical aspects of CDSS, but are not responsible for their contents.* (IT staff) | *I think accessing evidences in real time is extremely easy nowadays (…). The problem is not really accessing evidences, but how to select and apply them. It’s very important to consult with your colleagues, to have a good shared understanding of what a good and sound scientific evidence looks like. This all needs to be included in the […] process of developing CDSSs.* (Physician)  *We are in a hospital in which the “technological revolution” has been supported by the top management…. This was very important for the success of the CDSS. We managed to involve doctors in the process of selecting the content and the evidence-base of the CDSS… This allowed us to better understand the reasons why, initially, some clinicians didn’t trust it and did not want to use it.* (IT staff) |
| **4** | *Maybe I could use it. I think it would be more useful for young physicians, those who have only just graduated, or those with little experience... You know, to avoid mistakes...* (Physician)  *One problem may be the lack of evidence in the nursing field. CDSSs seem more useful to doctors than to us*. (Nurse) | *It’s brilliant. Really, really useful. I think it’s more for medics though, rather than for us (surgeons). When we have to make complicated decisions we very rarely have scientific evidences that can guide us. (For example) a raptured femur, should we use a nail or a plate? In the literature you find 5000 articles but just not the summary a CDSS could provide. […] I think physicians would find it very useful*. (Surgeon)  *CDSSs are extremely interesting in general medicine; it may be more difficult to build a system like this one for surgery specifically. Naturally, surgeons would benefit from using this system in the post-surgical care, especially with old and multi-pathologic patients... But I can predict that they would be less interested than medical doctors.* (Hospital manager) | *Any innovation that has the potential to affect clinical autonomy and decision-making shouldn’t be introduced like an imposition. If it’s perceived as a top-down order, clinicians will reject it. Physicians must agree and engage in the project. Perceive as something useful for them.* (IT staff)  *What was really key for us was working with clinicians to convince them that [CDSSs] are a tool for them. […] Something that could help them doing their job better, providing better care. […] We worked very hard to get them on board, to enthuse them and get them to contribute to this project. Some of them were excited about this from the start, some less so…* (Hospital Manager) | Not available |
| **5** | Not available | Not available | *I read a lot of oncology literature but there may be other areas that I’m less familiar with – I don’t know, cardiology for example. Most of our patients have multiple pathologies. It’s such a great help, having a system that provides us with the guidelines we need, when we need them, tailored to a specific patient.* (Physician)  I think [the CDSS] is an invaluable system. The only doubt that I had is whether… How to be sure that the alerts are updated in real time… That we do not have some messages, some alerts, that just sit in the system for two years while the guidelines change… (Nurse)  *It’s been a good experience so far. We’ve had a lot of support from the medical directors and some of the early-adopters in the wards […] Because when we first tested [the CDSS] the doctors didn’t like… they thought [CDSS’s alerts] were not right, the timing didn’t work. So we worked together to fix it and I think it’s working much better now… The doctors use it autonomously without asking anything to us, which usually means that things are working well.* (IT staff) | *We hear things like “The CDSS is just a stupid machine that cannot give me orders!” Well, I think the CDSS is indeed a stupid machine, but that stupid machine can sometimes be crucial to avoid mistakes. It may well happen that the stupid machine does not have all the elements to understand a specific case, but that’s OK, it’s our job to make decisions. If you know the evidences, you can decide not to comply with them if you have a good reason.* (Physician)  *Prescribing is the doctors’ role, but we are not only there to administer the drugs… If we have a doubt about a prescription, we do ask questions […] and most doctors are OK with this, they don’t perceive this as a challenge. […] If they say, you know, ‘I understand why you’re asking this, but in this case this is the right thing to do’, then I’m happy with it.[…] The CDSS helps because we are not always familiar with the drugs we administer; it is a quick way to double-check that everything looks OK with the prescription.* (Nurse)  *We worked very hard in the preparation phase to minimize the risk of failure. […] You need to enforce it but also to listen carefully to what your staff is saying… get a sense of the sentiment within your staff. You need to have systems in place to correct the CDSS in a timely way if something is not working.* (Hospital manager) |
| **6** | Not available | Not available | After we integrated the electronic record we started thinking about how the CDSS could do more than just reporting and actually help us in our practice. For example: if I prescribe an antibiotic for pneumonia, this will never be for less than a few days. […] We can teach the system to give me an alert only when a given time expires. (Physician)  *I think [the CDSS] saves time and energy... It helps the team to make faster decisions on therapies, and maybe starting treatment earlier… Being more effective really. […It’s] a really positive thing.* (Nurse)  *I would love to start thinking about decision support systems for our strategic and managerial choices too, evidences for decision-makers. We are really interested in evidence-based policy, how to use resources in a responsible and sensible way… We do a lot of work on this but I think we could improve in the way we access this knowledge on a day-to-day basis… The work on CDSS is inspiring us to improve, I think.* (Hospital manager) | *In the long term the introduction of the CDSS could have positive cultural and educational effects. It could help us developing an evidence-based culture within hospital teams. We could use it… Not just individually, but as a tool to get together and discuss complex cases and to monitor some particular patients.* (Nurse)  *I think it is important to create a multi-professional working group that meets regularly (…) All team members gather information from their colleagues and then feed it back into the group, so that we then adjust and shape the [CDSS] in a way that is consistent with clinicians’ needs*. (Hospital manager)  *One of the best outcomes we had from the implementation of CDSS is a stronger collaboration and alliance between the IT staff, on one side, and clinicians and the hospital management, on the other. (…) We felt that we were part of every important decision, we were actually part of the implementation team*. (IT staff) |
